# Assessment of patient follow-up from student-run free eye clinic to county ophthalmology clinic

**DOI:** 10.1038/s41598-022-05033-0

**Published:** 2022-01-19

**Authors:** Melanie Scheive, Lucas W. Rowe, Hanna L. Tso, Patrick Wurster, Nicholas E. Kalafatis, David A. Camp, Chi Wah Rudy Yung

**Affiliations:** 1 Department of Ophthalmology, Indiana University School of Medicine, 1160 W Michigan St., Indianapolis, IN 46202 USA; 2 Department of Ophthalmology, Baylor College of Medicine, 6565 Fannin St., Houston, TX 77030 USA

**Keywords:** Public health, Population screening

## Abstract

The Ophthalmology Student Interest Group at Indiana University School of Medicine provides a free student-run eye screening clinic for an underserved community in Indianapolis. Patients with abnormal findings are referred to the ophthalmology service of the local county hospital for further evaluation. This retrospective chart review studied 180 patients referred from our free eye clinic to follow up at the ophthalmology service of a local county hospital from October 2013 to February 2020. This study investigated factors impacting follow-up of patients by analyzing demographics, medical history, insurance coverage, and final diagnoses at follow-up. Thirty-five (19.4%) of 180 patients successfully followed up at the local county hospital with an average time to follow-up of 14.4 (± 15.9) months. Mean patient age was 51 (± 13.6) with nearly equal numbers of males and females. The most common diagnoses at follow-up included refractive error (51.4%), cataract (45.7%), and glaucoma (28.6%). Patients with diabetes diagnoses or Healthy Indiana Plan insurance coverage had increased probability of follow-up. This study reveals gaps in timely follow-up to the local county hospital, demonstrating the current limitations of our free clinic in connecting patients to more definitive care and the need for an improved referral process.

## Introduction

Preventive eye care is essential for preventing blindness and visual impairment. The utilization of such care depends on both the prevalence of eye conditions and patients’ ability to access care. However, access to timely ophthalmic care is particularly challenging for the low-income population who are uninsured or underinsured, which increases the likelihood of irreversible ocular impairment^1,2^. Targeted screening programs can provide opportunities to make timely diagnoses of ophthalmic conditions in patients with risk factors such as advanced age, hypertension, and diabetes mellitus.

Findings from existing ophthalmic screening programs, especially among underserved, low-income communities, suggest that follow-up rates are relatively low with some additional loss of adherence for patients who did follow up^3–6^. Similar dermatology screening programs indicate that most patients are unable to receive timely follow-up care^7^. Potential barriers to healthcare access include socioeconomic, geographic, and cultural factors^8^. Previously suggested solutions to improve follow-up include better access to transportation and enhanced patient education^4,5,9^. Furthermore, communication with patients without a stable phone number remains a challenge^5^.

The focus of this study is the ophthalmic visual screening program of the Indiana University Student Outreach Clinic (IUSOC), which is an interdisciplinary student-run free clinic that provides services in medicine, pharmacy, law, social work, dentistry, physical therapy, occupational therapy, women’s health, and nursing to an underserved population in Indianapolis, Indiana (IN). The Eye Clinic is run in partnership with the IUSOC and the Department of Ophthalmology at Indiana University School of Medicine (IUSM).

At the IUSOC Eye Clinic, patient visits began with collection of patient demographic information and medical history, followed by vision screening performed by medical students. Supervision was provided by IUSM ophthalmology residents and attending ophthalmologists. Eye examinations included distance visual acuity (VA) using a Snellen Chart, near visual acuity (NVA) using an Optec 5500 vision screener, visual fields (VF) using a Zeiss Humphrey FDT 710 visual field analyzer, refraction using a Topcon RM-8800 auto refractometer, intraocular pressure (IOP) using a Mentor Tono-Pen XL tonometer, fundus photography using a Centervue DRS non-mydriatic fundus camera, and slit lamp examination using a Topcon SL-3D.

Patients with concerning ophthalmic findings were referred for further evaluation, diagnosis, and treatment at the eye clinic of Sidney & Lois Eskenazi Health Services, a county hospital in downtown Indianapolis, IN. Referred patients were provided with the clinic contact information to independently schedule a follow-up appointment. The student-run free eye clinic did not directly schedule or coordinate follow-up appointments.

The purpose of this study was to evaluate the follow-up of patients referred from a high-volume student-run free eye clinic to the ophthalmology service of the local county hospital. Metrics included the follow-up rate, average time to follow up, and patient characteristics in this population, including demographics, medical history, insurance coverage, and final diagnosis at follow-up.

## Methods

Patients requiring referral to the ophthalmology service of the local county hospital after being examined at the IUSOC Eye Clinic between October 2013 and February 2020 were identified and included in the data analysis. Institutional review board (IRB) approval for this study was obtained through the Indiana University (IU) IRB. All methods were performed in accordance with these IRB guidelines. Informed consent was obtained in accordance with IRB requirements.

Data on demographics, insurance coverage, and medical history were retrieved from PracticeFusion (Practice Fusion Inc., San Francisco, CA), the electronic medical record (EMR) used by IUSOC. Data on follow-up to the local county hospital and diagnosis at follow-up were retrieved from Epic (Epic Systems Corporation, Verona, WI), the EMR used by Eskenazi Health Services. De-identified data were compiled and analyzed by the authors in a protected Excel (Microsoft Corporation, Seattle, WA) spreadsheet following the Health Insurance Portability and Accountability Act of 1996 policies on electronic protected health information.

A multivariate regression analysis was completed using follow-up as the binary dependent variable, with age, sex, ethnicity, insurance coverage, diabetes, and hypertension as the independent variables. One regression was performed with insurance coverage as a binary variable, and one was performed with specific insurance plans. The specific insurance plans included none, Medicare; Medicaid; the Healthy Indiana Plan (HIP), a unique Indiana Medicaid program; private insurance; and other. Statistical analysis was performed using IBM SPSS software (version 24.0, SPSS Inc., Chicago, IL, USA).

## Results

Between October 2013 and February 2020, 180 patients were referred from the IUSOC Eye Clinic to the eye clinic of Sidney & Lois Eskenazi Health Services. Thirty-five (19.4%) of these patients successfully followed up, with an average time to follow-up of 14.4 (± 15.9) months. Table 1 compares the demographic characteristics and medical histories of the referred patients who did or did not ultimately follow up.

**Table 1:** Follow-up and no follow-up patient demographics.

Demographic variable	Follow-up	No follow-up
Patients, *n*	35	145
Mean age ± SD, years (range)	53 ± 11.3 (21–77)	50.5 ± 14.1 (6–76)
> 65 years old, *n* (%)	5 (14.3%)	15 (10.3%)
**Medical history, *n* (%)**		
Diabetes only	9 (25.7%)	12 (8.3%)
Hypertension only	5 (14.3%)	45 (31.0%)
Diabetes and hypertension	6 (17.1%)	17 (11.7%)
**Insurance, *n* (%)**		
Healthy Indiana Plan	3 (8.6%)	2 (1.4%)
Medicare	2 (5.7%)	12 (8.3%)
Medicaid	3 (8.6%)	21 (14.5%)
Private insurance	0 (0.0%)	3 (2.1%)
No insurance	17 (48.6%)	62 (42.8%)
Other insurance	3 (8.6%)	7 (4.8%)
Not reported	7 (20.0%)	38 (26.2%)
**Sex, *n* (%)**		
Female	15 (42.9%)	68 (46.9%)
Male	15 (42.9%)	66 (45.5%)
Not reported	5 (14.3%)	11 (7.6%)
**Ethnicity,** ***n* (%)**		
African American	9 (25.7%)	50 (34.5%)
Asian	0 (0.0%)	2 (1.4%)
Caucasian	4 (11.4%)	25 (17.2%)
Hispanic	8 (22.9%)	21 (14.5%)
Other	1 (2.9%)	7 (4.8%)
Not reported	13 (37.1%)	40 (27.6%)

**Figure 1:**
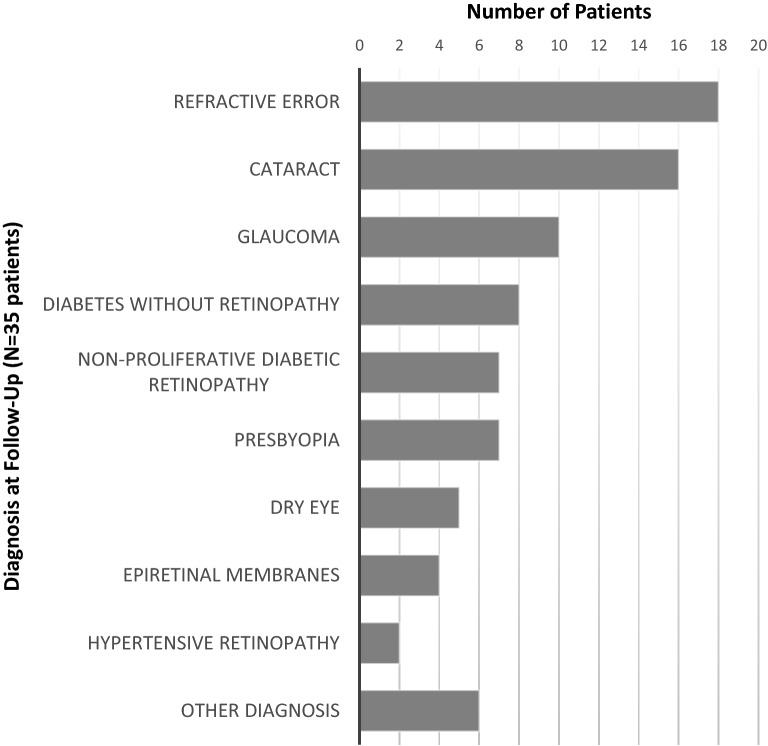
Summary of patient diagnoses at follow-up. The diagnosis or diagnoses of each patient at the follow-up appointment are listed. The other diagnosis category includes diagnoses that were only seen in one patient at follow-up. These diagnoses include central retinal vein occlusion, chorioretinal scar, ocular foreign body, macular lesion, posterior capsular opacification, and trichiasis.

Figure 1 presents the distribution of diagnoses at follow-up of the 35 patients who presented to the local county hospital. Each patient may have had one or more recorded diagnoses. The most common diagnosis was refractive error in 18 (51.4%) patients. Diagnoses under refractive error included astigmatism, hyperopia, and myopia. Presbyopia is listed separately as this diagnosis does not always result in net refractive error. Sixteen (45.7%) patients were diagnosed with cataract. Ten (28.6%) patients were diagnosed with glaucoma, including four patients who identified as African American, three patients between 41 and 50 years old, five patients between 51 and 60 years old, and one patient between 61 and 70 years old. Seven (20.0%) patients with diabetes were diagnosed with non-proliferative diabetic retinopathy, while the other eight (22.9%) patients with diabetes were not found to have diabetic retinopathy. Seven (20%) patients were diagnosed with presbyopia, including three patients between 41 and 50 years old, two patients between 51 and 60 years old, and two patients between 61 and 70 years old. Five (14.3%) patients were diagnosed with dry eye, four (11.4%) with epiretinal membranes, and two (5.7%) with hypertensive retinopathy. The following conditions were diagnosed in one (2.9%) patient each: central retinal vein occlusion, chorioretinal scar, ocular foreign body, macular lesion, posterior capsular opacification, and trichiasis.

Two multivariable regression analyses were performed using follow-up as the binary dependent variable, and age, female gender, ethnicity, insurance coverage, diabetes, and hypertension as the independent variables (Tables 2 and 3). Age was included as a continuous variable, with female gender, diabetes diagnosis, and hypertension diagnosis included as binary variables. Ethnicity was included as a categorical variable with patients who did not report an ethnicity as the reference category. Insurance coverage was included as a binary variable in the first regression, and as a categorical variable in the second regression with patients who did not report insurance coverage as the reference category.

**Table 2:** Results of regression performed with insurance coverage as a binary variable.

	Odds ratio	p-value
Age	1.02 (0.98–1.07)	0.34
Female gender	1.54 (0.53–4.47)	0.42
**Ethnicity**		
African–American	0.79 (0.23–2.68)	0.70
Caucasian	0.22 (0.03–1.44)	0.11
Hispanic	1.21 (0.28–5.24)	0.80
Asian	0.00	1.00
Other	0.00	1.00
Insurance	1.52 (0.51–4.59)	0.45
Diabetes	4.71 (1.42–15.58)	0.01*
Hypertension	0.35 (0.10–1.24)	0.10

*Reaches statistical significance (p < 0.05).

**Table 3:** Results of regression performed with specific insurance plans.

	Odds ratio	p-value
Age	1.05 (1.00–1.10)	0.07
Female gender	1.76 (0.60–5.15)	0.30
**Ethnicity**		
African–American	0.76 (0.23–2.55)	0.65
Caucasian	0.12 (0.01–1.03)	0.05
Hispanic	0.87 (0.20–3.76)	0.85
Asian	0.00	1.00
Other	0.00	1.00
**Insurance**		
None	1.66 (0.29–9.59)	0.57
Medicaid	2.35 (0.28–19.55)	0.43
Medicare	0.55 (0.04–8.00)	0.66
Private	0.00	1.00
Other	4.39 (0.39–49.82)	0.23
Healthy Indiana Plan	26.28 (1.63–422.80)	0.02*
Diabetes	5.84 (1.72–19.83)	< 0.01*
Hypertension	0.39 (0.11–1.34)	0.14

*Reaches statistical significance (p < 0.05).

The regression performed with insurance coverage as a binary variable revealed that a diabetes diagnosis was associated with a statistically significant increased probability to follow-up (OR, 4.71; 95% CI 1.42–15.58; p-value, 0.01). In comparison to patients who did not report an ethnicity, patients who reported their ethnicity as either African American, Asian, Caucasian, Hispanic, or other all revealed insignificant associations with follow-up (Table 2). Age, female gender, insurance coverage, and a hypertension diagnosis also revealed insignificant associations.

Similarly, the regression performed with specific insurance plans revealed that a diabetes diagnosis was associated with a statistically significant increased likelihood of follow-up (OR, 5.84; 95% CI 1.72–19.83; p-value < 0.01). Furthermore, in comparison to patients who did not report on insurance status, those with HIP insurance had a significantly increased likelihood of follow-up (OR, 26.28; 95% CI 1.63–422.80; p-value, 0.02). In comparison to patients who did not report on insurance status, all other insurance coverages revealed insignificant associations with follow-up (Table 3). In comparison to patients who did not report an ethnicity, patients who reported their ethnicity as African American, Asian, Caucasian, Hispanic, or other all revealed insignificant associations with follow-up. Age, female gender, and hypertension diagnosis also revealed insignificant associations.

## Discussion

Our free eye clinic has played an important role in the Indianapolis community by triaging basic eye complaints and connecting patients with ocular pathology to advanced ophthalmic care. Although our clinic provides basic vision screening and diagnostic services, as it is not equipped to treat advanced pathology, the referral process to the local county hospital is a crucial avenue for directing patients to necessary follow-up care. Indeed, the demographics of the patients who were referred and who ultimately received follow-up demonstrate the prevalence of pathology in this underserved Indianapolis community (Table 1). Thus, our free clinic serves as an initial triage point for patients who otherwise might be hesitant to seek care due to financial barriers or are unsure how to connect with ophthalmology specialty services.

This study demonstrated that our free eye clinic at the largest student-run clinic in the nation connected approximately one-fifth of patients to advanced ophthalmic care at the local county ophthalmology clinic. Thus, despite the identification of patients in need of further evaluation, the majority of patients did not ultimately follow up. The average time to follow-up was substantial at 14.4 (± 15.9) months. A significant contributing factor may be the qualifications for free eye care at our referral site. Eskenazi Health Services is a safety-net county hospital serving residents of Marion County. To receive free medical care from the hospital, patients must be county residents and have a total household income of less than twice the federal poverty line. Qualified patients are not required to have any other private health care insurance, but they may have concurrent Medicaid or Medicare coverage. For those who do not qualify, the hospital provides a free financial counseling service to help patients apply for medical coverage such as Medicaid, HIP, or other plans through the Indiana Healthcare Marketplace.

At follow-up, the most prevalent diagnoses were refractive error (51.4%), cataract (45.7%), and glaucoma (28.6%). Of the patients diagnosed with glaucoma, all patients with reported ethnicity were African American (40%), while most (90%) were over 40 years old. Given that glaucoma can lead to irreversible blindness, the prevalence of glaucoma diagnoses among our referrals underscores the important service our community clinic plays in screening and connecting such patients to crucial long-term care. Moreover, our clinic serves a patient population with many elderly African American individuals who are especially at risk for developing glaucoma^10^.

Diabetes diagnosis was associated with a statistically significant increased probability of follow-up (p < 0.01), possibly due to greater familiarity with the disease translating to an understanding of the importance of follow up. HIP insurance coverage was also associated with significantly increased likelihood of follow-up (p = 0.02), possibly due to patients’ familiarity with seeking health services at the county hospital with this insurance coverage and the county hospital’s acceptance of this insurance.

Our study has a number of limitations. The retrospective study revealed significant incomplete documentation of patient histories and encounters, affecting the data collection and analysis by underreporting. Furthermore, the accuracy of the data was likely limited by the reliance on self-reported history from patients. Documentation of the history, insurance coverage, screening findings, and examination results also likely differed among the medical students performing the encounters. Finally, although our study measured follow-up rate and average time to follow-up, we did not investigate factors that could influence timely follow-up. Future studies, such as patient surveys, could help to elucidate such barriers.

As the first longitudinal study of patient follow-up to a local county hospital in this underserved Indianapolis population, our findings have revealed the need for a better system for patients to access follow-up ophthalmic care. As with previous reports, our study further attests to the difficulty that low-income, uninsured or underinsured patients face in receiving timely ophthalmic care^3–7,9^. Innovative solutions will be needed to reduce the risk of irreversible ocular impairment in these vulnerable populations.

Despite providing contact information to refer patients from our free eye clinic to the local county ophthalmology clinic, our limited follow-up rate of 20% is due in part to patients’ challenges with scheduling and attending future appointments. Although not specifically addressed in this study, barriers to follow up in this vulnerable patient population are likely multifactorial, including lack of patient insurance coverage, financial stress, transportation difficulties, language barriers, limited health literacy, and lack of support in navigating the healthcare system to schedule follow-up appointments. Regarding insurance coverage, 44% (79/180) of referred patients reported that they had no insurance, with the true figure likely being higher given that an additional 25% (45/180) did not report their insurance status. The socioeconomic barriers to care cannot be understated in this inner-city Indianapolis population. Furthermore, patients residing outside the county may not have qualified for free care for non-urgent conditions at the county hospital. Patients with incomes too high to qualify for free care but too low to afford insurance may have been deterred by out-of-pocket expenses. Finally, patients with insurance coverage may have chosen to follow up with their own eye care provider rather than one at the county hospital eye clinic.

Similar follow-up barriers have been reported by other free ophthalmology clinics in underserved, socioeconomically vulnerable communities. For patients requiring specialized ophthalmic care, one student-run free clinic in Pittsburgh not only provided patients with the referral clinic contact information, but also provided the referral clinic coordinators with the patient contact information^11^. The coordinators then directly contacted the patients to schedule follow-up and assist with navigating programs such as Medicaid at no cost to the patient. This assistance resulted in a high follow-up rate of 72%. In their study, the follow-up rate was found to be lower among patients from communities located further from the referral clinic, possibly due to transportation barriers. Similarly, the geographical distance between our student-run free clinic and the county hospital clinic may have negatively impacted the follow-up rate.

In addition, a student-run free eye clinic in San Francisco serving the local homeless population found that patients without a primary care physician or high school diploma were less likely to follow-up at the local county hospital for advanced ophthalmic care^12^. This free clinic scheduled follow-up appointments for a specific date and time. This study attributed their limited follow-up rate of 41% to multiple barriers preventing the homeless population from accessing the healthcare system, including social isolation, transient living situations, limited health literacy, and mistrust of the healthcare system. As our Indianapolis clinic’s intake form does not inquire about homeless status, our study did not evaluate the unique barriers to care in this subset of underserved patients.

Despite our clinic’s vital service to the Indianapolis community, more work remains to be done to improve the referral follow-up rate and decrease the average time to follow-up. One recent avenue for improvement was the establishment of a Patient Navigator (PN) system for the free eye clinic in November 2020. The goal of the PN system was to increase follow-up communication with patients seen at the free clinic to ultimately connect more patients with advanced care. Under this system, medical student volunteers known as Patient Navigators (PN’s) contact patients requiring further care after their in-person free eye clinic visit to ensure they are able to schedule a follow-up appointment at the local county clinic. Thus, our PN’s serve a similar role as the referral clinic coordinators in Pittsburgh^11^. Previously, our free clinic would provide patients with instructions to call the county clinic to make an appointment. The results of this study encompass data from before the initiation of the PN system. Under the new system, patients are given the same instructions to call the county clinic but are additionally contacted by PN’s who can provide support throughout the process, including reminding the patient to call, coordinating with the county clinic, and offering assistance to apply for Medicaid.

Furthermore, PN’s now assist in scheduling patients for initial appointments at our free eye clinic. As our eye clinic is one of many services offered by the IUSOC, many patients are referred to the IUSOC Eye Clinic from other services such as the IUSOC Medicine Clinic for diabetic or hypertensive retinopathy screenings. Patient navigators call these patients to schedule them for the next free eye clinic date. Previously, our clinic operated entirely on a walk-in basis. The new scheduling system is an important step forward given that our clinic only operates on one Saturday per month. Patients are contacted and informed of a specific date and time that they can show up to receive basic eye care. Thus, the PN system plays a new vital role in establishing both an inflow and outflow of patients to and from our clinic.

To further improve the patient referral experience, we hope to enlist PN’s in surveying patients whom they contact to elucidate barriers to follow-up. This information would be valuable in providing further context for the results of this study and initiating quality improvement projects to support patient follow-up. For future studies, we hope to compare the patient follow-up rates before and after implementation of the PN system to demonstrate the efficacy of this novel referral process. Such improvements will ultimately help patients in this vulnerable Indianapolis population more easily receive the ophthalmic care they need.
